# Comparative Analysis of the Biomechanical Behaviour of Two Cementless Short Stems for Hip Replacement: Linea Anatomic and Minihip

**DOI:** 10.1371/journal.pone.0158411

**Published:** 2016-07-08

**Authors:** Sergio Gabarre, Antonio Herrera, Elena Ibarz, Jesús Mateo, Jorge Gil-Albarova, Luis Gracia

**Affiliations:** 1 Department of Mechanical Engineering, University of Zaragoza, Zaragoza, Spain; 2 Aragón Institute for Engineering Research, Zaragoza, Spain; 3 Department of Surgery, University of Zaragoza, Zaragoza, Spain; 4 Aragón Health Sciences Institute, Zaragoza, Spain; 5 Department of Orthopaedic Surgery and Traumatology, Miguel Servet University Hospital, Zaragoza, Spain; University of Zaragoza, SPAIN

## Abstract

A comparative study between two stems (Linea Anatomic and Minihip) has been performed in order to analyse the differences in their biomechanical behaviour, concerning stem micromotions and load transmission between stem and bone. From the corresponding finite element models, a parametric study was carried out to quantify ranges of micromotions taking into account: friction coefficient in the stem-bone interface, press-fit and two types of gait cycle. Micromotions were evaluated for each stem at six different levels along repeated gait cycles. An initial and marked stem subsidence at the beginning of the simulation was observed, followed by an asymptotic decrease due to friction forces. Once migration occurs, a repeated reversible cyclic micromotion is developed and stabilized as gait cycle times are simulated. The general motion pattern exhibited higher amplitude of micromotion for Minihip compared to Linea stem. The load transmission mechanism was analyzed, identifying the main internal forces. The results show higher local forces for Minihip stem up to 80% greater than for Linea stem. The differences of design between Minihip and Linea conditioned different distributions of load, influencing the posterior stress-shielding. Consequently, short stems require high bone stock and quality should, being indicated for young patients with high bone quality.

## Introduction

The modern age of total hip arthroplasty (THA) began in the 60’s with the advent of low friction arthroplasty designed by Charnley [1]. The main goals of hip arthroplasty are to reduce pain, to improve articular performance and guarantee the implant’s survival in the long term. During the past five decades, there has been a significant evolution in design, bearing materials and fixation systems [2].

Cementless hip arthroplasty emerged at the end of the 70s, as an alternative to cemented systems, for younger patients and higher mobility demands. Continued changes in design and in materials have been made in order to improve osseointegration of components and guarantee long-time survival. One of the main problems with cementless stems is achieving a good primary fixation which permits the implant’s osseointegration and thus good long term outcomes, since primary fixation is crucial to promote bone ingrowth. Additionally other key points are reducing stress-shielding, which happens to either a higher or lower extent in every cementless stem with less intensity in cemented stems, and preserving the maximum bone stock in case of potential arthroplasty revisions. The age of clinical indications for total hip arthroplasty has changed over the last few years towards younger patients with higher demands on daily activity levels [3].

Short femoral stems constitute one alternative which has emerged in recent years, minimizing stress-shielding and preserving bone-stock for future revisions [4–9]. A controversial issue is whether short stems can maintain stability in the long term, which is essential for prosthesis survival [7, 10–12]. The implant’s primary stability is mandatory to achieve osseointegration, which is essential for the survival of every cementless implant. An early stem migration is the preamble of aseptic loosening [13, 14]. Several factors account for primary stability such as implant size and design, level of press-fit achieved, bone quality, avoiding gap between bone and implant and the patient’s weight [15]. All the aforementioned factors are important to minimize micromotions between the implant and bone. Although no acceptable magnitude is known as to the micromotions needed for osseointegration [15], a micromotion amplitude lower than 150 μm is accepted [16].

Regarding finite element (FE) simulation works, shape optimization studies [17, 18] were developed focusing on the design of cementless stem geometry in terms of primary stability. Kadir and colleagues [19] studied micromotions at two points (distal and proximal) and the effect of press-fit for an uniform interference for an Alloclasic hip stem comparing simulation results to in vitro measures. Finally, Monea studied the insertion process during press-fit for three stem designs in terms of residual stress and contact distribution [20]. Despite the above studies, a controversial issue is whether short stems can provide primary stability in the post-operatory, essential to achieve osseointegration, which is needed for the stability in the long term and then the prosthesis survival.

The aim of this work is to perform a numerical comparative study, by means FE simulation, between two types of short stems: the Linea Anatomic (Tornier, Montbonnot Saint Martin, France) and the Minihip (Corin-Group, Cirencester, United Kingdom), with which we have experience with a mean clinical follow-up of 5 years with good results, in order to analyse the differences in their biomechanical behaviour, with regard to stem micromotions and load transmission between stem and bone. Moreover the objective is to support the clinical study based on the mechanical behaviour of the stems. To this respect a parametric study concerning friction coefficient in the stem-bone interface, press-fit and two types of gait cycle was performed.

## Materials and Methods

**Fig 1:**
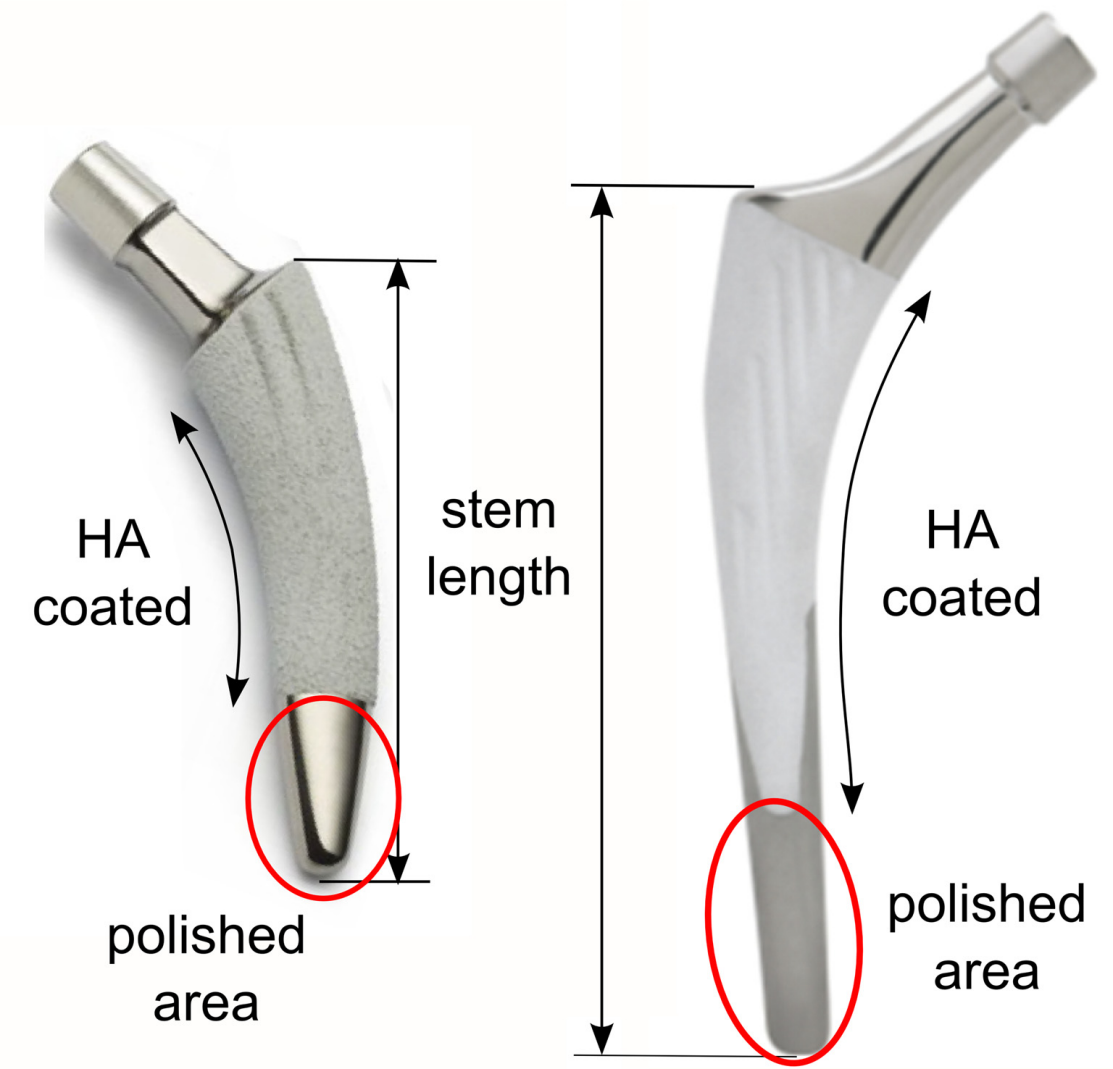
Stems. (A) Minihip stem. (B) Linea stem.

The Minihip stem has an anatomic shape following the natural curvature of the medial calcar, preventing breach of the greater trochanter. It highly conserves bone, retains femoral neck and leaves diaphysis intact. This stem has a low profile neck and taper to maximize head/neck ratio and consequently, maximize the safe range of the implant´s motion without compromising its fatigue strength. The coating of the stem represents 80% of its surface and the length thereof is between 79.5 and 117.5 mm depending on the size of the stem (Fig 1A).

The Linea anatomical stem design is supposed to achieve a good primary anchoring in the axial plane thanks to its double cone design and articular geometry reproduction. It is completely coated at the metaphysis level and partially coated at the diaphysis by rugged titanium and hydroxyapatite (HA) having an striated surface; only at the antero-internal and postero-external sides, limiting coating in loaded zones, the surface coating of the stem is approximately 75% and the length thereof is 100 to 140 mm (Fig 1B).

Sizes elected for the simulation were the No. 3 in both stems, because they are the most commonly used in the clinical practice in our hospital. The length of Mini-Hip is 89 mm and Anatomic Linea is 110 mm. Although both stems belong to the short stem family, the Linea Anatomic is a stem with a geometrical design similar to conventional long stems which has a shortened length. The conventional cementless stems have lengths between 140 and 170 mm.

A parametric study was carried out to quantify ranges of micromotions and load transmission between stem and bone, taking into account different variables: friction coefficient in the stem-bone interface, press-fit and two types of gait cycle.

**Fig 2:**
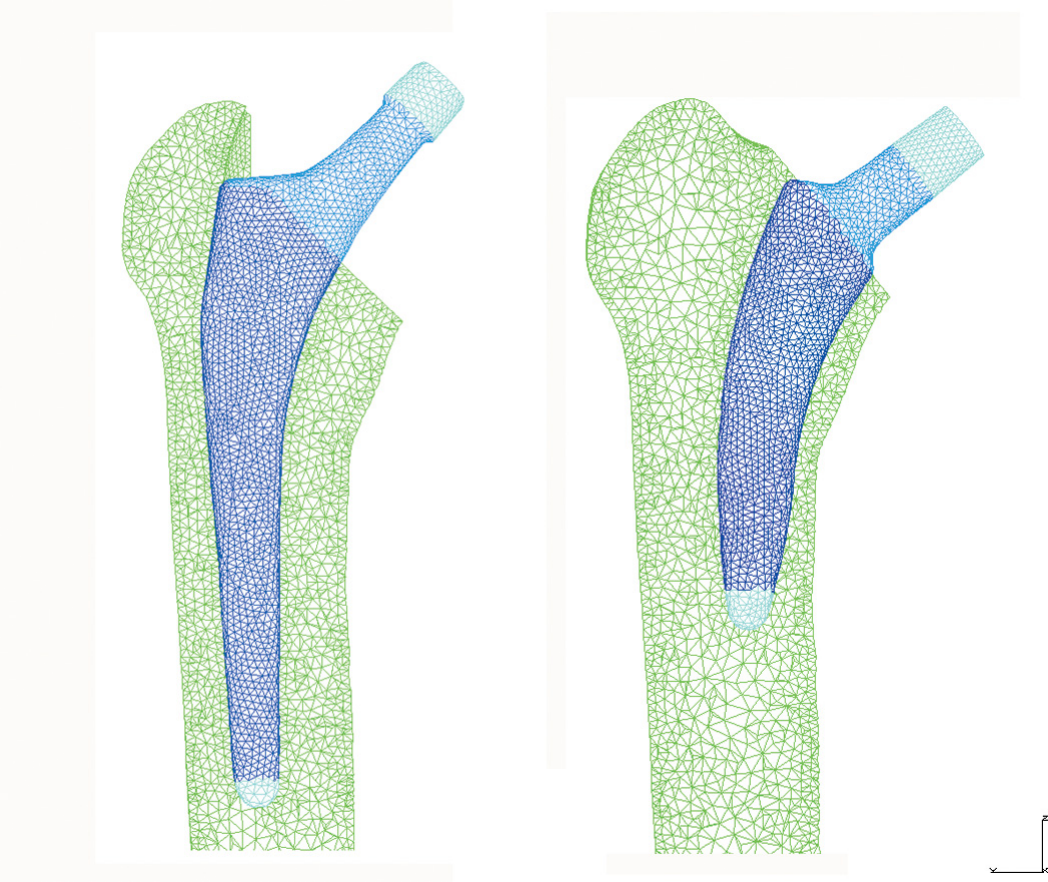
FE model of both implanted stems. (A) Linea stem. (B) Minihip stem.

For that purpose, two finite element (FE) models were developed, corresponding to each type of stem implanted in a whole femur belonging to a 35 year old donor, as it represented an indicated bony geometry to implant both short stems. The bones of cadaver donor were provided by the Official Bone Bank, featuring all authorizations of the National Organization of Transplants. A three dimensional (3D) scanner (Roland® PICZA Irvine, California) with a resolution of 0.2x0.2 mm. was used to obtain the geometry of the femur and both stems. After modelling the femur, the stems were placed in the same position as they would be in real surgery. With regard to element type selection and the minimum mesh size required, a mesh refinement was performed to achieve a convergence towards a minimum of the potential energy, with a tolerance of 1% between consecutive meshes. I-deas® 11 NX Series PLM software (Siemens, Plano,Texas) was used for meshing and Abaqus 6.12 software (Dassault system’s Simulia corp, Providence, Rhode Island) was used for calculations and post processing. In both models, tetrahedral elements with quadratic approximation (C3D10 in Abaqus nomenclature) were used. The model for the Linea stem consisted of 395909 elements for bone and 31162 elements for stem, while the model for the Minihip stem consisted of 393744 elements for bone and 22773 elements for stem (Fig 2). The material of both stems was titanium alloy (Young’s modulus: 110316 MPa; Poisson ratio: 0.3).

A computed tomography (CT) scan (512x512 acquisition matrix, field of view (FOV) = 240 mm, slice thickness = 0.5 mm, in plane resolution) was obtained using a TOSHIBA Aquilion 64 scanner (Toshiba Medical Systems, Zoetermeer, Netherlands). Based on the CT images from the donor, mechanical properties of each element of the bone mesh were assigned. By segmenting the CT images in Mimics 15.0 software (Materialise Leuven, Belgium), Houndsfield Units (HU) of each node were converted to r_app_ by linear relationships as follows [21]:

- Proximal femur:

$${\text{r}}_{\text{app}}=\;131\;+\;1.067\;\text{CT}\#$$ (1)

- Distal femur:

$${\text{r}}_{\text{app}}=\;139\;+\;1.205\;\text{CT}\#$$ (2)

where: r_app_ (kg/m^3^) is the apparent density and CT# are HU. Young’s modulus *E* (MPa) was calculated accordingly to the following equations:

- Diaphyseal femur [22]:

$$\text{E}\;=\;2875\;{\text{r}}^{3}{}_{\text{app}}$$ (3)

- Metaphyseal femur [23]:

$$\text{E}=\;1000\;(-13.43\;+\;14.\;261\;{\text{r}}_{\text{app}})$$ (4)

**Fig 3:**
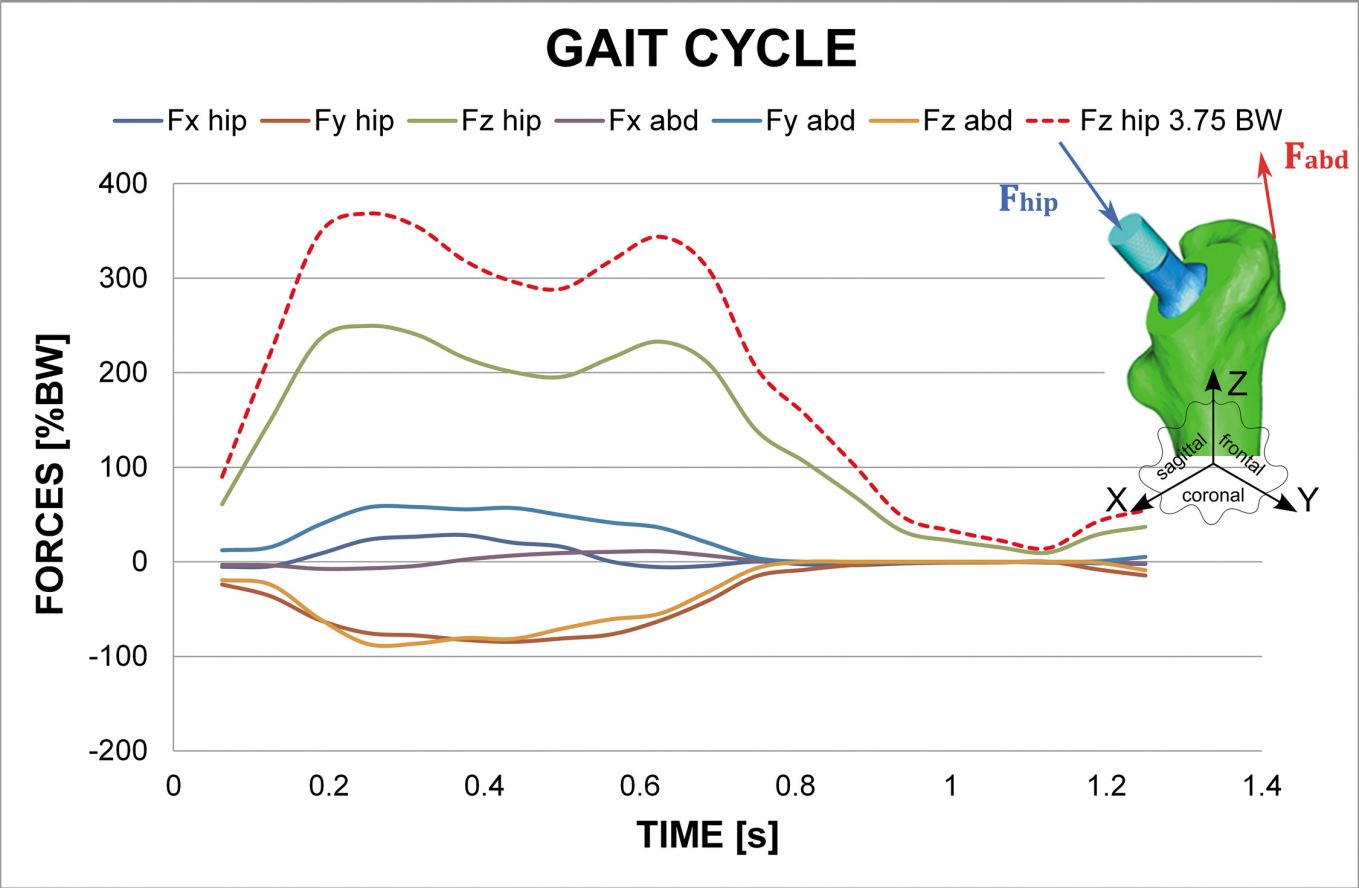
Hip reaction forces and abductor muscle forces values during gait cycle corresponding to a normal gait cycle and a “vigorous” gait cycle.

The averaged Young’s modulus value for each element and the HU value assignment to its constituent nodes was developed by an in-house algorithm, having maximum values of 20000 MPa and 960 MPa for cortical and trabecular bone, respectively. Poisson ratio was 0.3 both for cortical and trabecular bone. Anatomical boundaries between diaphysis and metaphysis were stablished in accordance to orthopaedic surgeon’s indications. Fully constrained boundary conditions were applied at the condyles. Orthoload’s database [24] was used to apply hip reaction forces at the head of the stem and adductor ones as the most representative muscle force. These varying loads during gait cycle were simulated as tabular-defined amplitude forces. Orthoload´s database provides mean values of muscle force and hip reaction along 20 frames for a time of 1.25 s gait cycle (Fig 3). As the intention was to study initial subsidence and micromotion stabilization, continued gait cycles were simulated as the initial transitory phase and the following stationary one were clearly defined. Two types of gait cycle (body weight (BW): 70 kg) were considered: the first one corresponds to “normal” gait cycle which Z component had a peak load of 2.54 BW at heel strike (referred as 2.54 BW), whereas the second one corresponds to a “vigorous” gait cycle associated to a heavier or more active patient which Z component had a peak load of 3.75 BW at heel strike (referred as 3.75 BW). The rest of load components are in accordance with the scaled Z component.

Loads are transmitted by contact interaction between the rough titanium proximal part and interface of the bone. The smooth and titanium-crafted, polished, distal part doesn’t have any relevance as the implant barely transmits loads in this part. This zone of interest is divided into six equally-spaced planes sketched by means of two auxiliary splines along the stem´s external and internal sides called plane cuts. Relative displacements were post-processed as the direct difference between coordinates in deformed configuration among corresponding nodes in stem and bone, respectively, along all the gait cycles simulated. To this respect, in the meshing process six spline curves of homologue pairs of nodes were defined equally spaced along the hydroxyapatite coated area, with a minimum of 12 nodes pairs at each curve.

Another variable considered is the friction coefficient for both stems, with values ranging from μ = 0.3 to μ = 0.6, which correspond to the range of values used by most of authors [25–27]. These values are illustrative and are based on the principle that only friction contact is simulated as interaction along the bone-stem interface. The dissipating effect of friction is appreciable but minor, selecting μ = 0.5 as the reference value for results presented, beyond which a stable behaviour was observed, with equivalent results for the relative movements. As the simulations carried out refer to postoperative period, before any biological process started, i.e. bone remodelling process, no tied constraints have been applied. Moreover, no contact was considered between stem and bone marrow because its stiffness is too low and both stems have rounded and polished tip.

The size of the last reamer corresponds with the size of the implant. Thus the implant serves itself as the operating last reamer before its virtual implantation. Once reaming is determined by the size of the implant, both stems are guided through the intramedullary femur canal, and inserted by impact at the top with a mallet. This procedure is quite unclear and relies on the surgeon’s experience and visual evaluation: a press-fit is yearned without risking fracturing the femur by excess of transversal stresses and seating the stem correctly. That procedure was simulated for both stems applying several increasing vertical loads at both stem impaction areas, studying 3 press-fit levels. Loads of 1.20, 2.40 and 5.00 kN, respectively, were applied to the Linea stem to attain the last press-fit. Following an analogous procedure, loads of 0.56, 1.12 and 2.60 kN, respectively, were applied to the Minihip stem. The press-fit levels were adjusted to obtain similar maximum circumferential stresses and contact pressures in both stems.

These increasing levels of load, although seemingly arbitrary, were similar to those reported in literature when investigating press-fit for acetabular cups [28] and within the range of three types of stem studied by Monea [20]. Press-fit was measured as the contact pressure at each stem level. This contact pressure increased as press-fit grew. Circumferential tension stresses were also recorded to verify if the operated femur integrity was safe when press-fitting was performed. In addition to this, fluxes associated to minimal and maximal principal stresses were analyzed in order to determine load transmission mechanism.

In summary, the following results will be processed and analyzed: circumferential stresses for different press-fit levels, micromotions evolution during gait cycle, general layout of 3D micromomotions, fluxes associated to minimal and maximal principal stresses and load transmission mechanism, considering eccentricities between load application point and mechanical axis of the femur.

## Results

**Fig 4:**
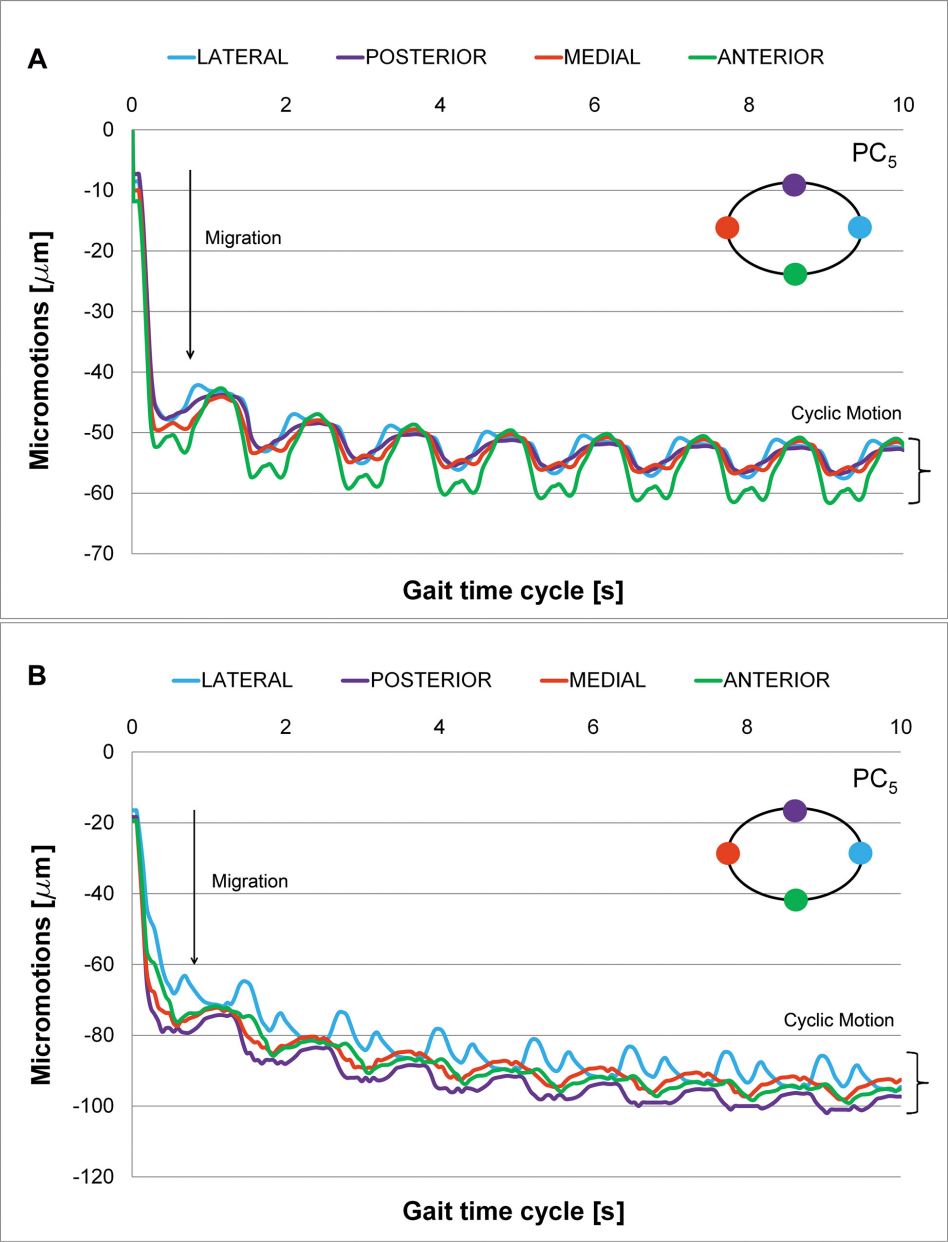
Micromotions along Plane Cut 5 (friction coefficient μ = 0.5). (A) Linea stem. (B) Minihip stem.

Tables 1 and 2 summarize the results of the simulation for each press-fit level for the Linea and Minihip stems, respectively in terms of circumferential tension stresses. Micromotions have been evaluated and post-processed at each stem level along repeated gait cycles. One initial and marked stem subsidence can be observed at the beginning of the simulation, referred to as “irreversible migration” (Fig 4). This subsidence is a permanent displacement of the stem from the initial unloaded situation. There is a sudden increase of migration, followed by an asymptotic decrease caused by the friction forces. Once migration occurs, a repeated “reversible cyclic micromotion” is developed and stabilized as gait cycle times are simulated. Cyclic micromotion amplitude is then measured once the stem has been stabilized. The initial transitory subsidence phase can be clearly distinguished, which little by little is followed by the cyclic stationary motion phase. Stationary cyclic motion is placed at the bottom of the graph as charts are grouped in a column that defines the amplitude of the micromotion.

**Table 1:** Press-fit level and circumferential stress for Linea stem at every plane cut.

Circumferential Stresses (MPa)	PRESS-FIT 1 (1.20 kN)	PRESS-FIT 2 (2.40 kN)	PRESS-FIT 3 (5.00 kN)
Plane Cut 6	29.68	52.76	111.09
Plane Cut 5	25.24	45.33	57.18
Plane Cut 4	14.32	26.04	53.68
Plane Cut 3	7.32	13.38	27.61
Plane Cut 2	7.91	14.29	29.34
Plane Cut 1	10.06	17.85	35.10

**Table 2:** Press-fit level and circumferential stress for Minihip stem at every plane cut.

Circumferential Stresses (MPa)	PRESS-FIT 1 (0.56 kN)	PRESS-FIT 2 (1.12 kN)	PRESS-FIT 3 (2.60 kN)
Plane Cut 6	28.99	48.44	121.09
Plane Cut 5	14.66	29.21	68.09
Plane Cut 4	7.09	14.34	33.41
Plane Cut 3	6.88	14.29	32.66
Plane Cut 2	6.02	12.13	28.92
Plane Cut 1	10.51	19.42	43.54

**Fig 5:**
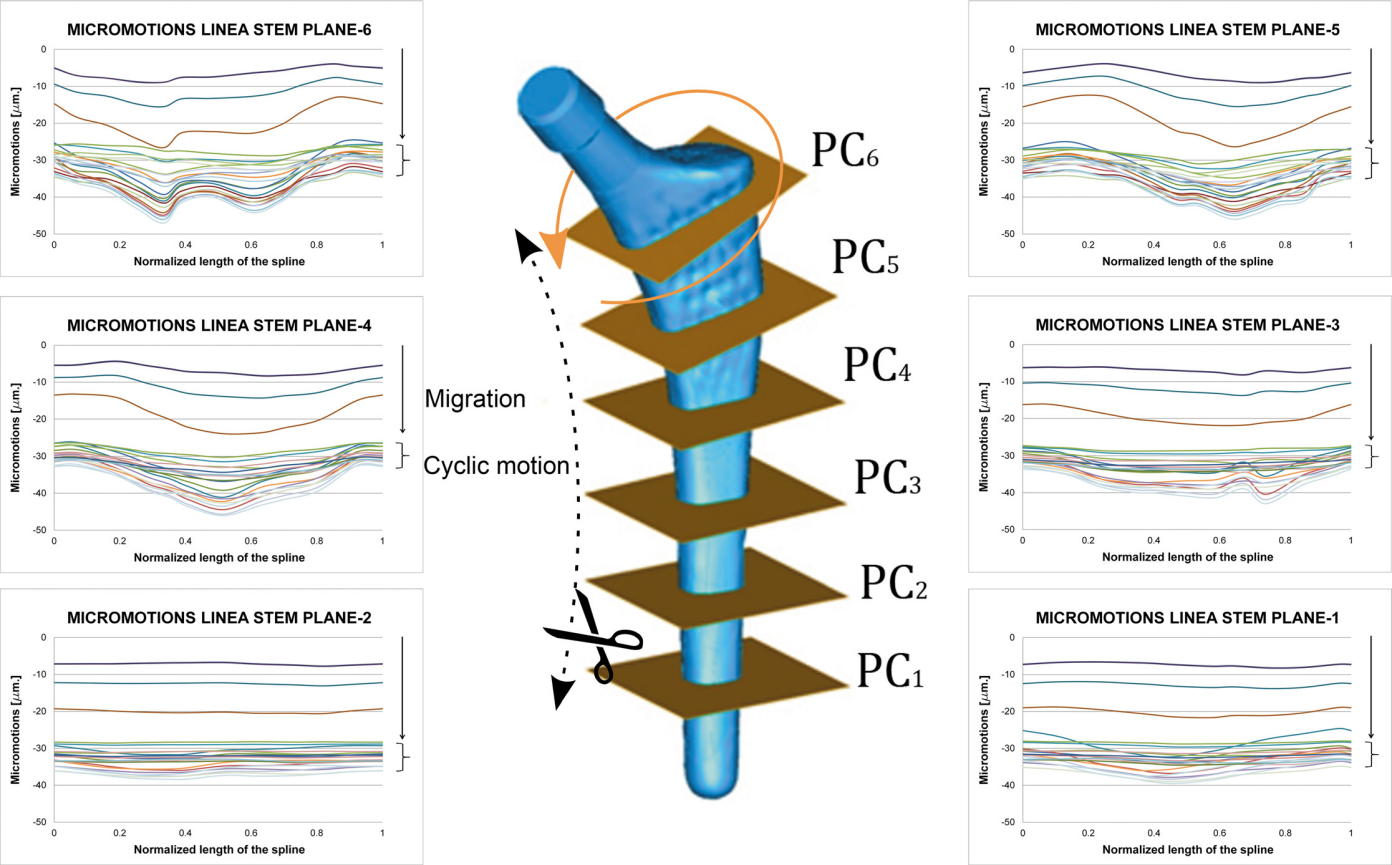
Micromotions along plane cuts for Linea stem (friction coefficient μ = 0.5). Normalized length in charts corresponds to the developed length for each plane cut starting at medial point in counter clock wise sense. Curves correspond to the different instants in gait cycle.

**Fig 6:**
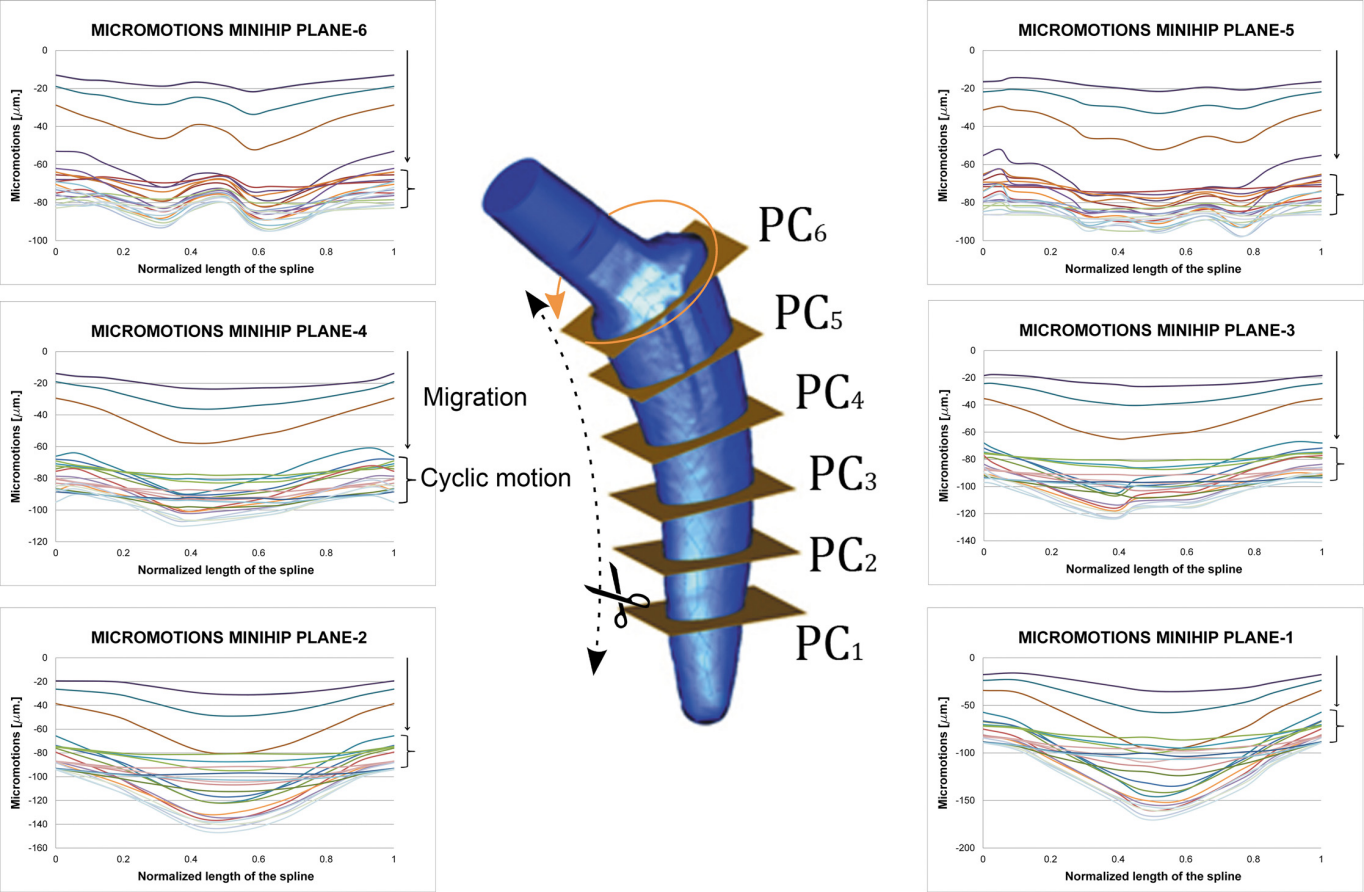
Micromotions along plane cuts for Minihip stem (friction coefficient μ = 0.5). Normalized length in charts corresponds to the developed length for each plane cut starting at medial point in counter clock wise sense. Curves correspond to the different instants in gait cycle.

Processing the results corresponding to each plane cut, 3D movement at the different analyzed levels can be deduced (plane cuts 1 to 6). Figs 5 and 6 give a general layout of this 3D movement at each plane level selected for the Linea and Minihip stems, respectively. The rate of the micromotions for the Minihip is higher than for the Linea stem. The highest amplitude is reached towards the bottom (first and second plane cut) for the Minihip. On the other hand the largest amplitude of micromotion occurs towards the top of the Linea stem (sixth and fifth plane cut). Statistical correlation between both stems in terms of cyclic micromotion was analyzed by means of Pearson R^2^ coefficient (R^2^ = 0.838 normal gait cycle and R^2^ = 0.775 for vigorous gait cycle, respectively). In both stems, beyond the initial irreversible migration, relative micromotions remain stable describing a stationary cycle (stacked up curves at the bottom of the charts).

**Fig 7:**
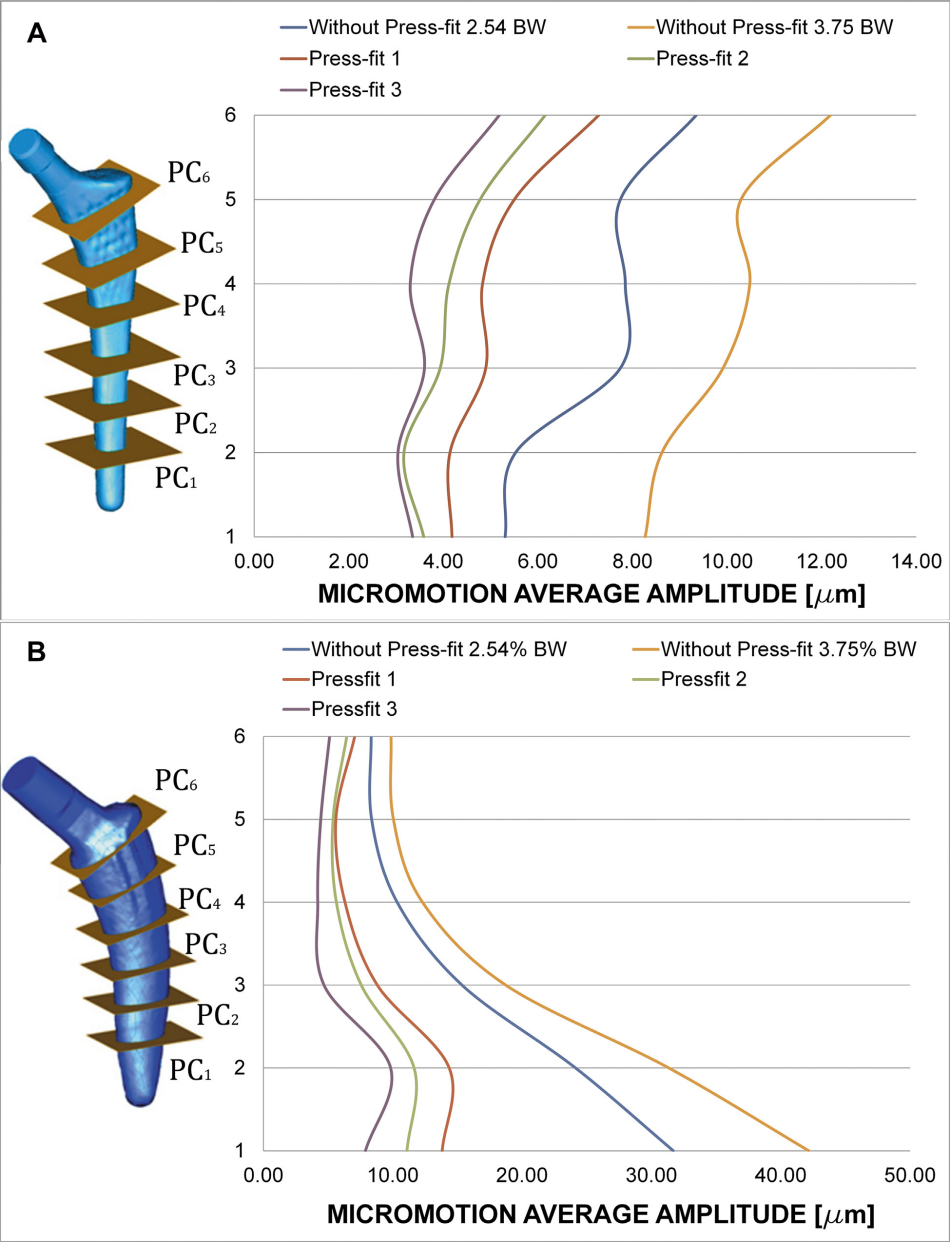
Average amplitude of micromotions for different press-fit levels and gait load cycle (friction coefficient μ = 0.5, Titanium alloy). (A) Linea stem. (B) Minihip stem.

Charts of Fig 7A and 7B show the effect of introducing press-fit. The average amplitude (mean value for the twelve homologue pairs of nodes at each plane cut) decreases at all the studied plane cuts for both stems. Amplitudes for all the plane cuts become closer: the behaviour for each stem is more uniform. The increase in the amplitude of load cycle yields to bigger micromotion amplitudes.

Press-fit is believed to stabilize cementless stems, limiting the relative motion at bone interface. Charts of Fig 7A and 7B clearly show this trend: average amplitude of cyclic motion diminishes as press-fit level increases. Another conclusion to point out is that the relative difference of amplitude along each plane is smaller when introducing press-fit. This effect can be related to the fact that press-fit introduces a compression state on the stem that makes it work less as a solid rigid, prevailing the complete elastic subsidence. Figures also show the increasing effect of amplitude as the load scenario becomes greater: a “vigorous” gait cycle increases micromotion amplitude.

Differences between press-fit levels are fairly similar, showing a very uniform behaviour along the stem. The general motion pattern exhibited higher amplitude of micromotion for the Minihip stem compared to the Linea stem. Conversely to it, micromotion increased towards the bottom of the stem. The increased load level of gait cycle led to similar results in the Linea stem: it augmented the amplitude. Press-fit levels decreased micromotion as the contact pressure generated grew. A similar effect was also observed here: micromotion levels tended to be more uniform between each other whenever press-fit was applied, the stem has a less solid rigid motion.

The influence of the coefficient of friction was also studied, although its dissipating effect was appreciable it was minor, in accordance with previous works [26, 29]. From friction coefficient value from 0.3 to 0.5, a noticeable decrease in micromotions is observed. Beyond a value of 0.5 a stable behaviour was observed, with equivalent results for the relative movements between stems and bone.

**Fig 8:**
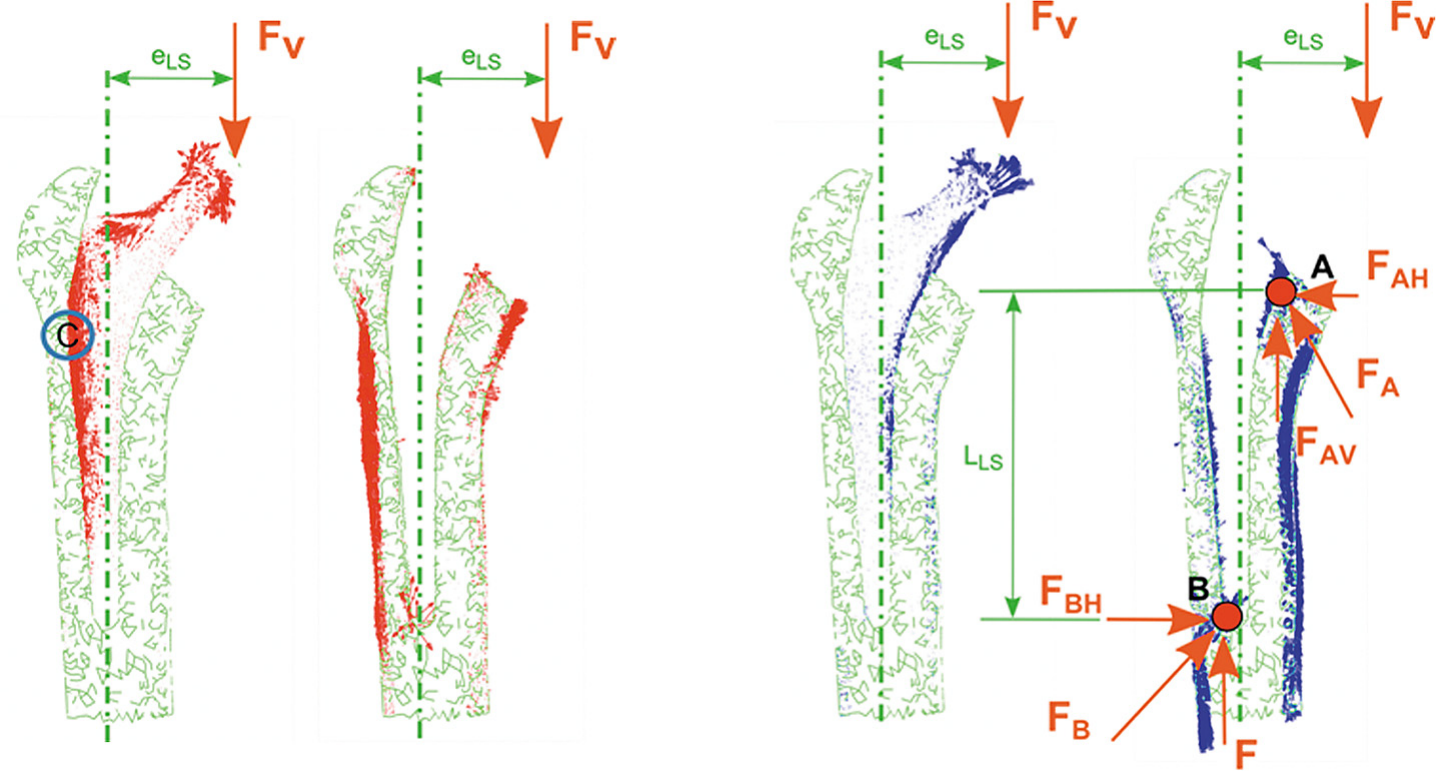
Coronal plane. **View of principal flux stresses. Proximal femur with/without Linea stem.** (A) Maximum principal stress. (B) Minimum principal stress.

**Fig 9:**
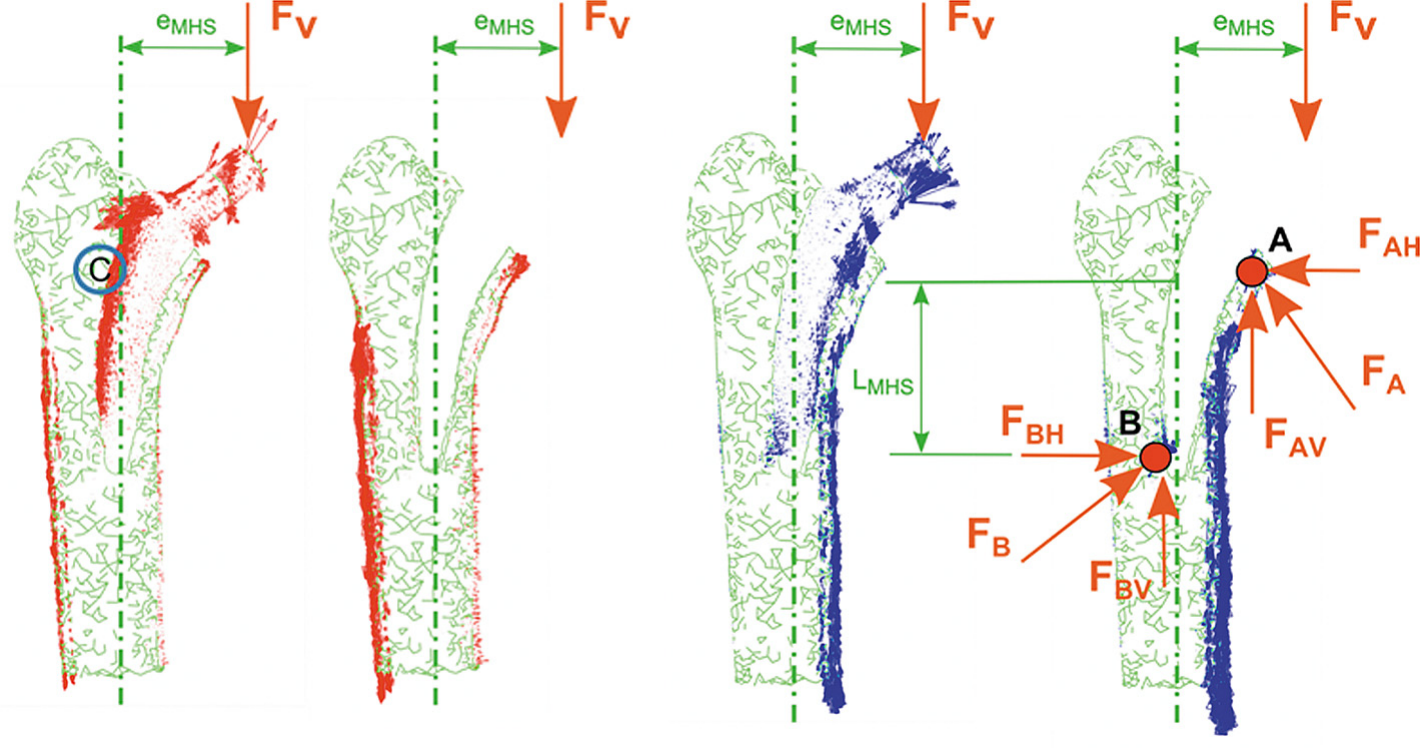
Coronal plane. **View of principal flux stresses. Proximal femur with/without Minihip stem.** (A) Maximum principal stress. (B) Minimum principal stress.

The load transmission mechanism between stem and bone depends on the eccentricities (e_LS_, e_MHS_) between load application point and mechanical axis of the femur (Figs 8A and 9A), yielding to a lever effect with an associated bending moment. This effect can be observed in Figs 8B and 9B.

Load transmission mechanism is based on compression stresses transmitted by contact: a primary compression and a secondary one, marked with A and B, respectively, in Figs 8B and 9B. Vertical components of primary and secondary compression resist the vertical load F, and the corresponding bending moment F*e is counterbalanced with the pair of horizontal forces F_AH_ and F_BH_. The latter forces equilibrate each other.

## Discussion

In 2006 was published the first work of simulation by FE to study the micro-movement between the bone and one short stem (Mayo) and the possibility of reducing the contact-stress in relation to the geometrical design and the extension coating of the stem [17].

Since 2006 to the best of our knowledge, the present work is the first that employs FE simulation to study the micromotions that occur at bone-stem interface of short cementless stems in the postoperative period; other studies by FE have been performed to study bone remodelling and stress-shielding after the implantation of short stem [30, 31]. The micromotions have also been studied in vitro [4, 5, 32].

Short stems have been introduced to preserve more bone stock, but have raised doubts as to whether they are able to maintain primary stability, essential to allow osseointegration and stability for ensuring the survival of the femoral stem, as an early migration leads to loosening [14].

Many factors influence primary stability: press-fit level, implant design, mechanical quality of patient’s bone, size of the implant, weight of the patient, or implant material [15]. Among all these factors, press-fit is believed to be the key one. Press-fit level remains uncertain: the surgeon is guided by visual and auditory clues when deciding if the implant is “firmly” seated. This variable, in practice, must lie within a range which limits micromotions and prevents the femur from being fractured by the stem. The amplitude of micromotions should be in the range 50–150 microns to achieve good osseointegration, higher amplitude of micromotions leads to the formation of fibrous tissue and future implant loosening [16].

In view of the obtained results, the average amplitude of micromotions for both stems lies below the aforementioned critical limits of 50–150 μm. All the average amplitudes corresponding to each plane cut lay below this critical threshold. Although micromotions at the first and second plane cut (out of the hydroxyapatite coated area) don’t play an important role in osseointegration, their amplitudes are post-processed in order to analyze the stem mechanics completely. Therefore, it can be concluded that the general mechanical performance of both stems is suitable for bone ingrowth. Studies in vitro performed with fresh human femurs have shown that short stems have an amplitude of micromotion less than 150 microns [5, 32], in accordance with our findings.

Despite a certain correlation between both stems in terms of cyclic micromotion is observed for the two types of gait cycle (R^2^ = 0.838 normal gait cycle and R^2^ = 0.775 for vigorous gait cycle, respectively), the mechanical behaviour is different: maximum displacement values are obtained at the metaphyseal zone in Linea stem whereas they are obtained at the diaphyseal zone in Minihip stem. The values are more uniform along the Linea stem.

The initial stem subsidence obtained at the beginning of the simulation has been reported by other authors in clinical studies [33, 34] and in other in vitro studies [4, 19, 35]. Results in terms of the averaged cyclic micromotion and initial migration are in accordance with Kadir [19], which used the Alloclassic stem in an “in vitro” study, ranging initial subsidence from 30 to 35 μm and the amplitude of the cyclic micromotion from 4 to 10 μm. However, the obtained results disagree with Fottner [35], which uses the Metha prosthesis and SUMMIT prosthesis, ranging initial subsidence from 290 to 430 μm and a cyclic micromotion from 60 to 80 μm for the first one, and with averages values of 400 μm of subsidence and 40 μm of cyclic micromotion, for the second one. On the other hand, Westphal [4] uses Proxima stem with subsidence ranging from 300 to 400 μm and with a mean cyclic amplitude of 50 μm. Both studies were realized using cadaveric samples. These comparisons are only qualitative, as prosthesis designs differ; size and age of donors, and loading tests vary. Other authors, in studies by means of radiostereometric analysis with tantalum markers, conclude that the subsidence in a short stem is less than in standard stems [11, 36].

With respect to load transmission, cementless stems transmit load through friction and pairs of opposite forces. Their design (especially marked for the Linea stem) exhibits a wide profile at the proximal area decreasing towards the bottom point. Although every manufacturer boasts that its commercial stems are purely metaphyseal, it is not totally true. In fact, the results show that a lever effect is produced in both stems with horizontally placed components, the lever effect being higher in the Minihip stem and producing high values of compressive stresses, both in the proximal and distal areas of the stem in contact with bone.

When studying the load transmission, it can be observed that the stem receives the hip load (main vertical component) at a distance (e_LS_, e_MHS_) from its longitudinal axis, yielding to a lever effect. That eccentricity produces a bending moment additional to the vertical load. Consequently tension stresses are developed along the outer side of the stem and compression ones through its inner side. During the very initial stage after implanting these cementless stems, no osseointegration is initiated. Therefore, contact surfaces are not able to transmit tension stresses because they would produce a contact loss. This effect can be observed at point C in Figs 7A and 8A: tension flux along the stem is interrupted at that location.

Load eccentricities are within the same range (e_LS_≈e_MHS_), but the Minihip stem has its compression zones A and B (Figs 7 and 8) closer to each other than the Linea stem. As a consequence lever arms are smaller, increasing horizontal components at the primary and secondary compression areas up to 80% with respect to the Linea stem. The surface in contact in the Minihip stem is lower than in the Linea stem, so vertical load resisted by contact is therefore lower, increasing also the vertical component at points A and B. Compression flux at distal part is more inclined in the Linea stem. This fact suggests that vertical and horizontal components at point B are nearly equal, while for the Minihip, they are almost purely horizontal. This assumption can also be applied for the proximal part. It can be noticed that the loading mechanism at the proximal part for the Minihip stem is different: it scarcely compresses the bone, suggesting that load at this point is resisted by circumferential traction stresses.

Consequently stress-shielding decreases from the Linea stem to the Minihip. The stem design influences the load transmission and therefore a greater or lesser stress-shielding, although in all short stems stress-shielding is lower than in conventional stems [10, 30, 37]. Other authors think that proximal load transmission is more physiological in the short stem, but stress-shielding remains in these models [32, 36, 38].

In light of these assertions, indications of use for each stem clearly match the clinical indications. High bone stock and bone quality are required to select a short stem, since loads transmitted to the bone are higher and surface in contact is smaller. This affirmation is linked to the fact that it’s indicated for young patients with high bone quality [7, 39], although good results have also been published in older people [40, 41].

In designs having more internal load transmission an obvious improvement in stress-shielding occurs in Gruen zone 7 [30, 37, 42]. Each design varies the stress-shielding and if this is less, it will have better adaptive remodeling and less loss of bone mass density (BMD) in the proximal femur [10, 43]. The surgical technique is very important, above all in the conservation of femoral neck and correct placement of the stem to obtain a good stem support in the proximal femur [4, 44]. However, a longer neck can condition limb length discrepancy [45]. In any case, preoperative planning and the choosing of the correct size of stem are necessary [46].

The present work confirms that the amplitude of micromotions in the postoperative period does not condition the secondary stability of short stems required for achieving osseointegration, as confirmed by other clinical works on patients who were operated with short-stems of different designs over several follow-up periods [6–9, 36, 40, 41, 47].

The length of the stem does not affect the stability [5, 48] of short stems if there is a good anchorage in the area metaphyseal-epiphyseal femoral. The literature has removed the doubts about stability in these stems [7]. A good primary stability allows for a good osseointegration. This leads us to conclude, in agreement with other authors, that short stems are an excellent surgical option for the treatment of osteoarthritis of the hip and osteonecrosis of the femoral head, especially in young people with good bone quality and functional high demand, with excellent results in the medium and long term [7, 40, 42, 44, 49–51]. That allows the patients to participate in several sports, except in high impact activities [52]. The implantation of a short stem is a technique that allows mini invasive surgery (MIS) and with less complications than in the implantation of standard prostheses [53, 54]. Although there is a 10 year follow-up period work is reported in the literature [8], future work should be carried out to validate, support and contrast the presented simulations with clinical evidence over a longer period of follow-up [55].

The observed biomechanical behaviour corroborates our clinical results, although they are referred only to a 5 year follow-up period. In conclusion, short stems are a good alternative to conventional stems in THA, being the Minihip the most advisable because its better load transmission.

## Limitations of the Present Study

There is no experimental validation, neither application of a cohort with different ages nor femur sizes. Only one cadaveric sample has been used in this study, which means that the effect of age, size, etc. was not taken into account. No biological effects (regeneration or bony ingrowth) were simulated. The relative motion magnitudes reported cannot be directly compared to in vivo magnitudes or relative interface motion values reported in the literature.

Concerning numerical simulations, the usual limitations inherent to this kind of studies are present. So, the bone was modelled in a simplified way as a linear elastic isotropic material, whereas its actual behaviour is close to an orthotropic material; although dynamic loading are applied to the implant in the actual gait cycle, only equivalent static loading was considered in this work. Finally, other physiological factors were not taken into account: age, gender and bone condition.

## Conclusions

With our work of simulation by FE we can deduce that the amplitude of micro-movements of short stems in the postoperative period are compatible with achieving osseointegration and secondary stability of the stem, in accordance with previously published studies using radiostereometric analysis, in vitro studies or clinical follow-up. However, it is clear that the clinical follow-up with short stems, on average 5 years in different published papers, is not enough and that new studies will be needed with more years of follow up to confirm the results obtained from the FE simulation in the present work.

## Supporting Information

S1 Fig **A and B. Stems**. (A) Minihip stem. (B) Linea stem.
(TIF)

S2 Fig **A and B. FE model of both implanted stems**. (A) Linea stem. (B) Minihip stem.
(TIF)

S3 Fig Hip reaction forces and abductor muscle forces values during gait cycle corresponding to a normal gait cycle and a “vigorous” gait cycle. (TIF)

S4 Fig **A and B. Micromotions along Plane Cut 5 (friction coefficient μ = 0.5).** (A) Linea stem. (B) Minihip stem.
(TIF)

S5 Fig Micromotions along plane cuts for Linea stem (friction coefficient μ = 0.5). Normalized length in charts corresponds to the developed length for each plane cut starting at medial point in counter clock wise sense. Curves correspond to the different instants in gait cycle.
(TIF)

S6 Fig Micromotions along plane cuts for Minihip stem (friction coefficient μ = 0.5). Normalized length in charts corresponds to the developed length for each plane cut starting at medial point in counter clock wise sense. Curves correspond to the different instants in gait cycle.
(TIF)

S7 Fig **A and B. Average amplitude of micromotions for different press-fit levels and gait load cycle (friction coefficient μ = 0.5, Titanium alloy).** (A) Linea stem. (B) Minihip stem.
(TIF)

S8 Fig **A and B. Coronal plane. View of principal flux stresses. Proximal femur with/without Linea stem.** (A) Maximum principal stress. (B) Minimum principal stress.
(TIF)

S9 Fig **A and B. Coronal plane. View of principal flux stresses. Proximal femur with/without Minihip stem.** (A) Maximum principal stress. (B) Minimum principal stress.
(TIF)

S1 Table Press-fit level and circumferential stress for Linea stem at every plane cut. (TIF)

S2 Table Press-fit level and circumferential stress for Minihip stem at every plane cut. (TIF)

## Abbreviations

THA: Total Hip Arthroplasty
FE: Finite Element
HA: Hydroxyapatite
3D: Three Dimensional
CT: Computed Tomography
FOV: Field of View
HU: Houndsfield Units
BW: Body Weight
BMD: Bone Mass Density
