# Supplementary figures and images for: Comparative Analysis of the Biomechanical Behaviour of Two Cementless Short Stems for Hip Replacement: Linea Anatomic and Minihip
Source: PLoS One. 2016 Jul 8;11(7):e0158411. doi: 10.1371/journal.pone.0158411 (PMC4938462; doi:10.1371/journal.pone.0158411)

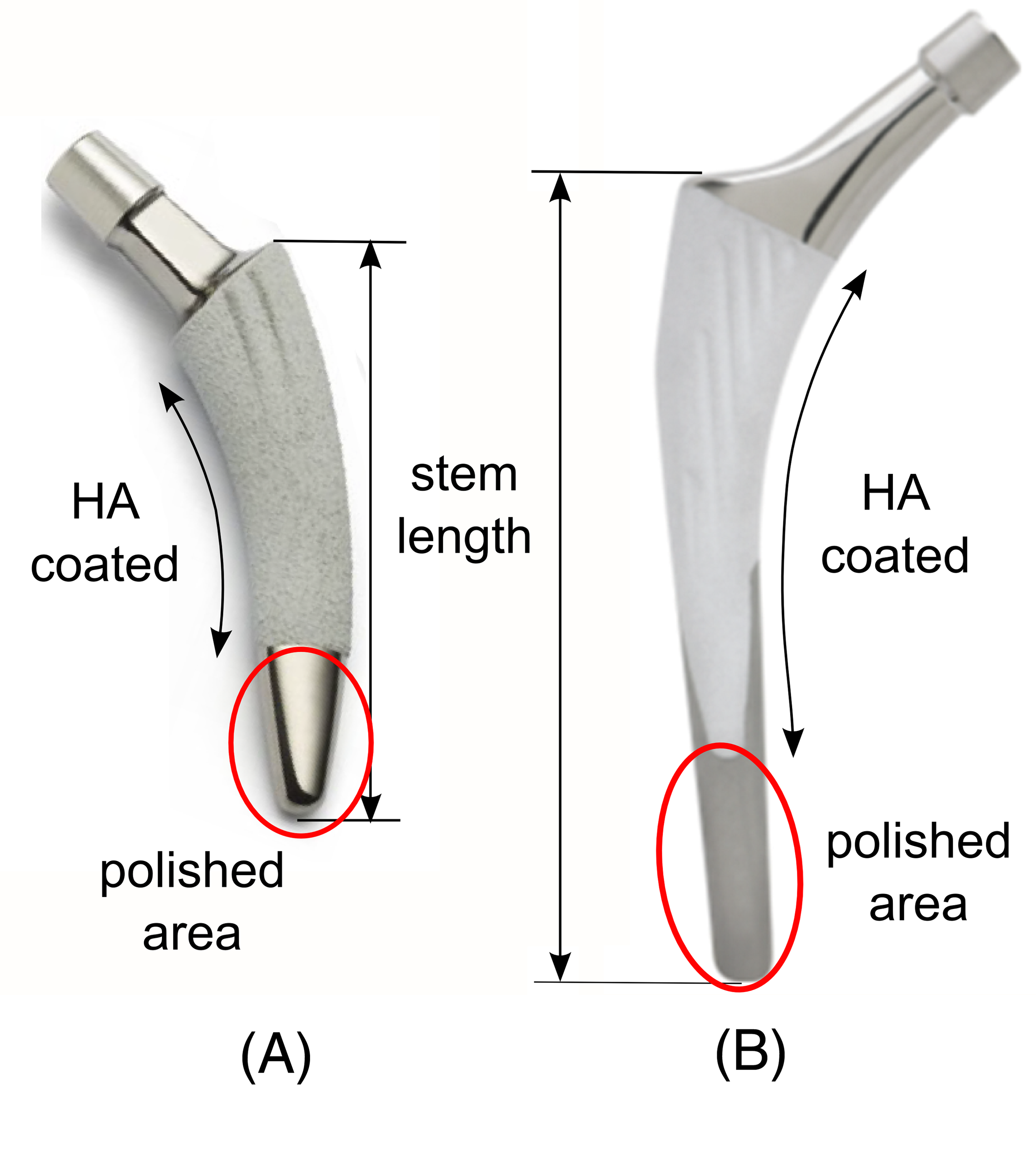

Supplement: S1 Fig — A and B. Stems. (A) Minihip stem. (B) Linea stem. (TIF) [file pone.0158411.s001.tif]

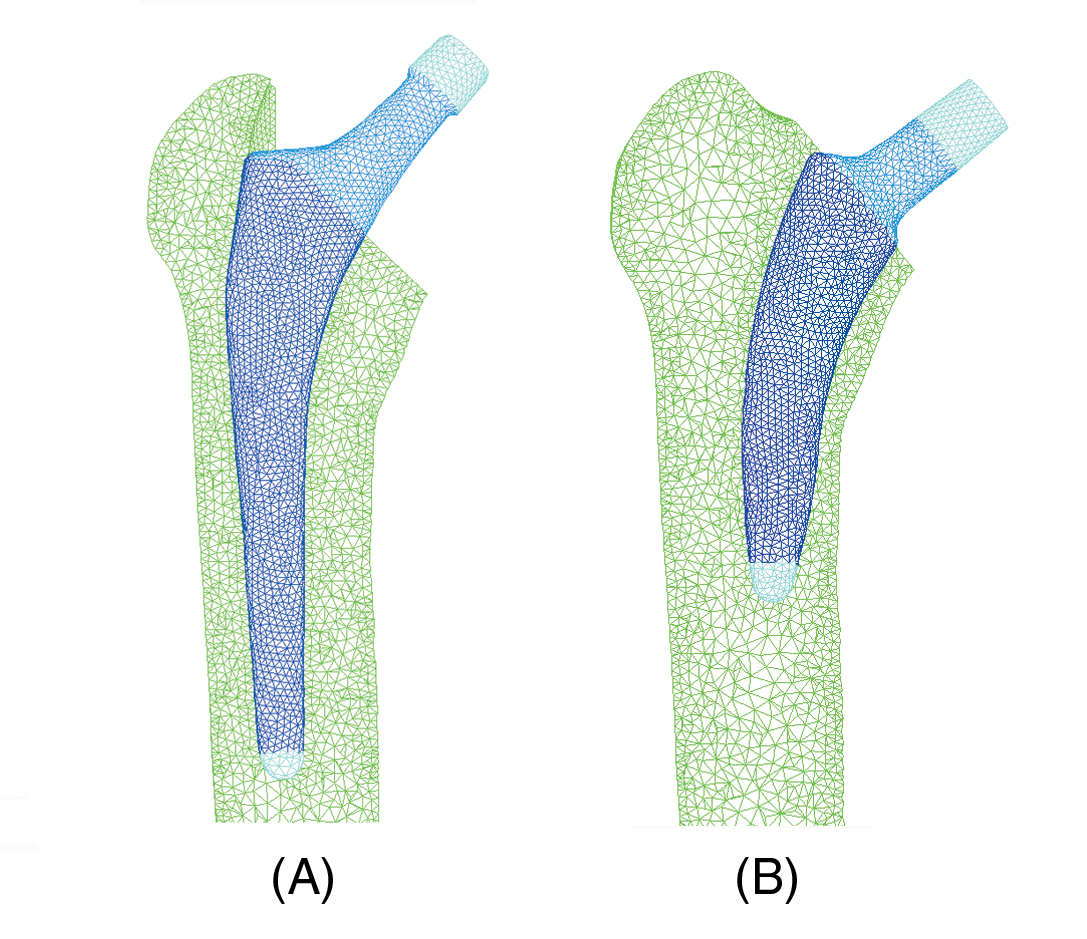

Supplement: S2 Fig — A and B. FE model of both implanted stems. (A) Linea stem. (B) Minihip stem. (TIF) [file pone.0158411.s002.tif]

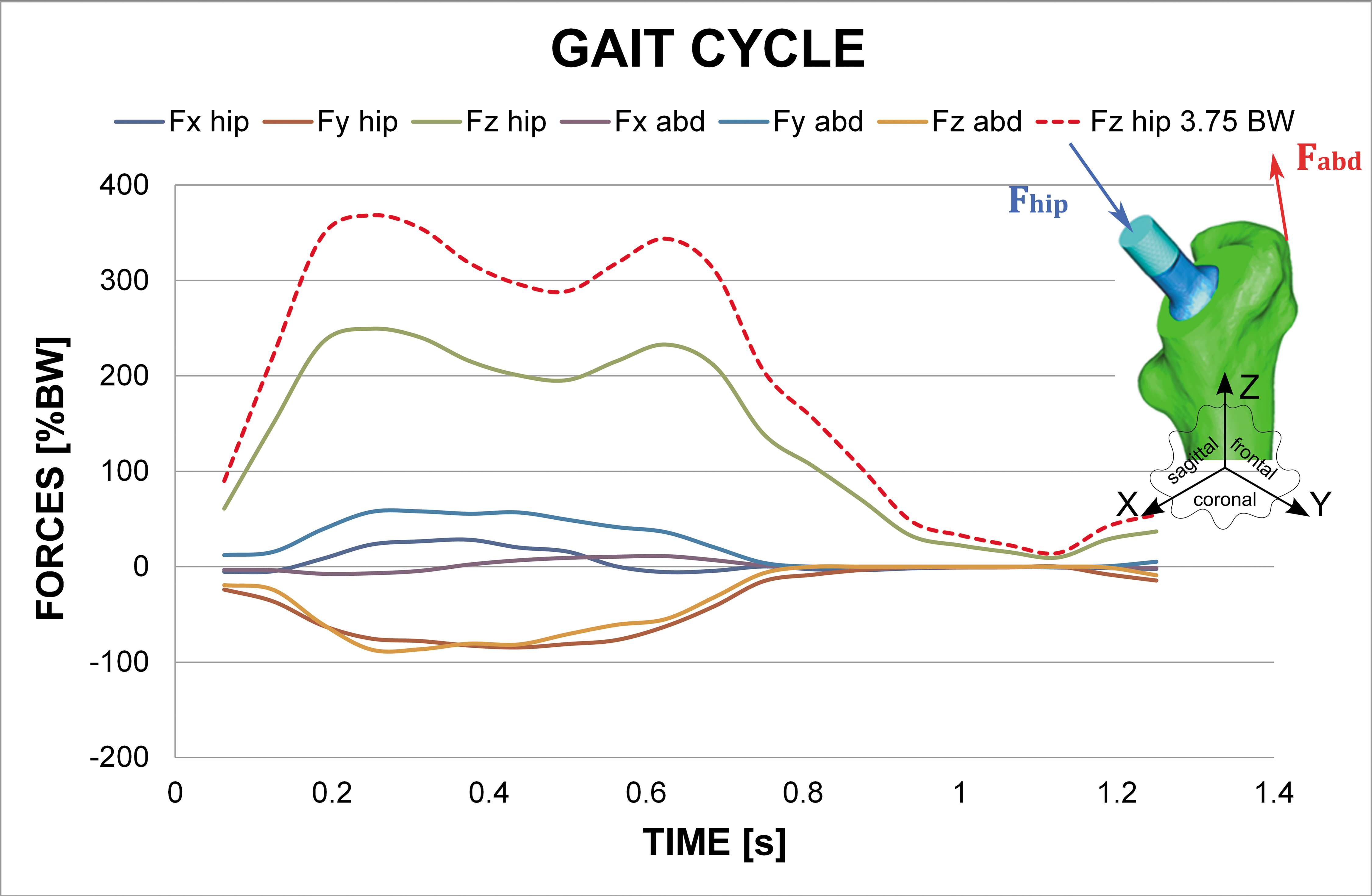

Supplement: S3 Fig — (TIF) [file pone.0158411.s003.tif]

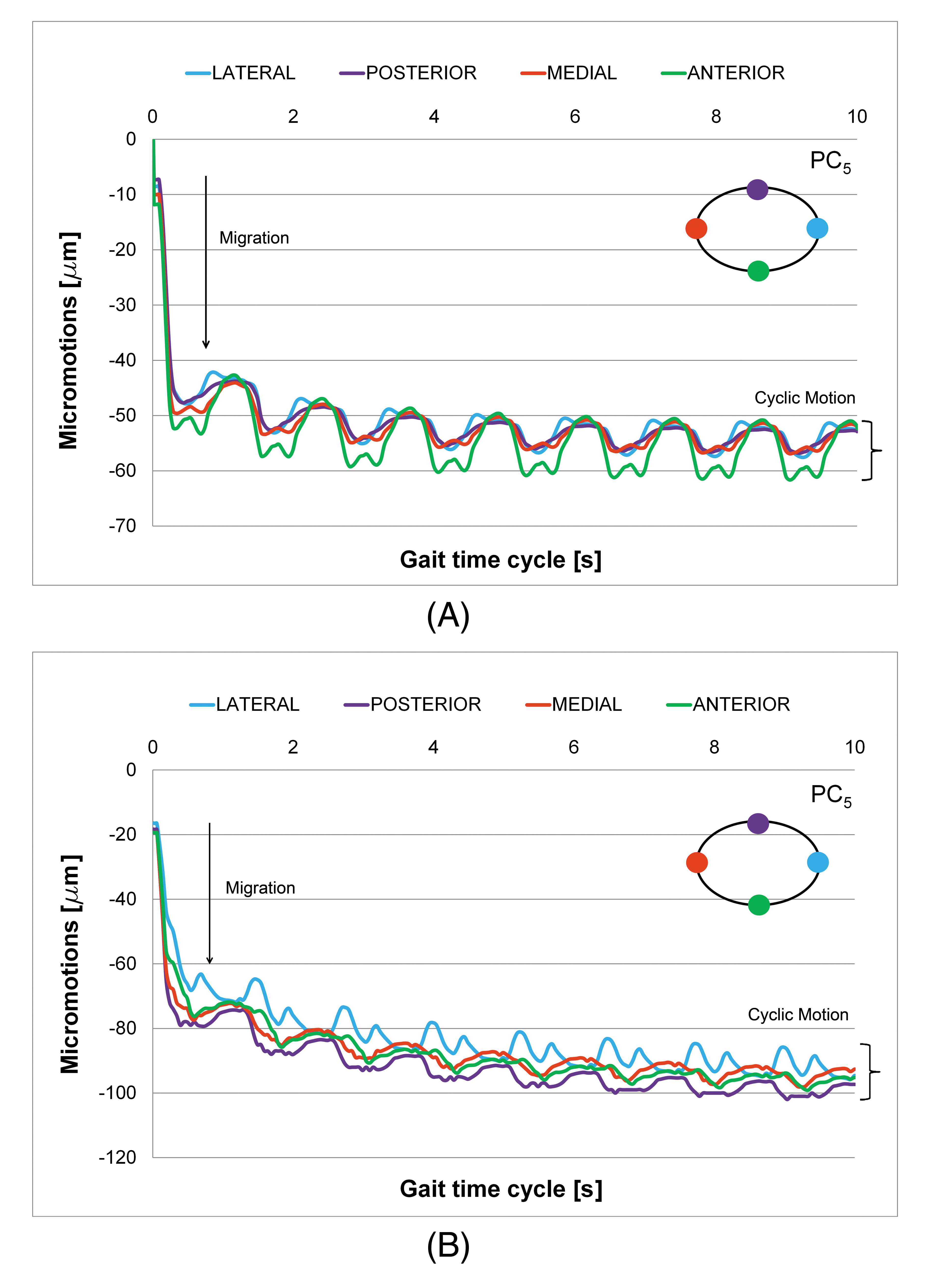

Supplement: S4 Fig — A and B. Micromotions along Plane Cut 5 (friction coefficient μ = 0.5). (A) Linea stem. (B) Minihip stem. (TIF) [file pone.0158411.s004.tif]

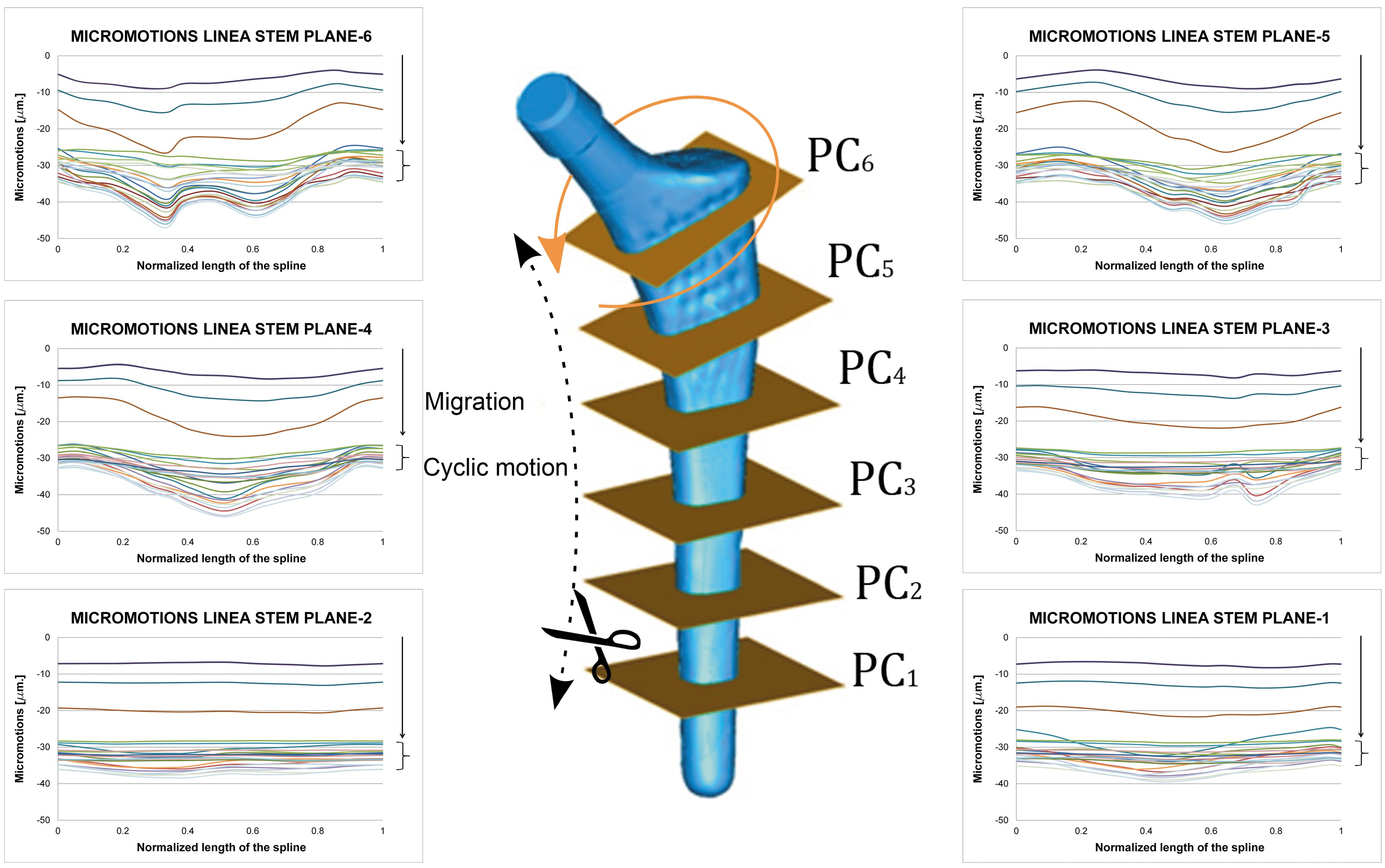

Supplement: S5 Fig — Normalized length in charts corresponds to the developed length for each plane cut starting at medial point in counter clock wise sense. Curves correspond to the different instants in gait cycle. (TIF) [file pone.0158411.s005.tif]

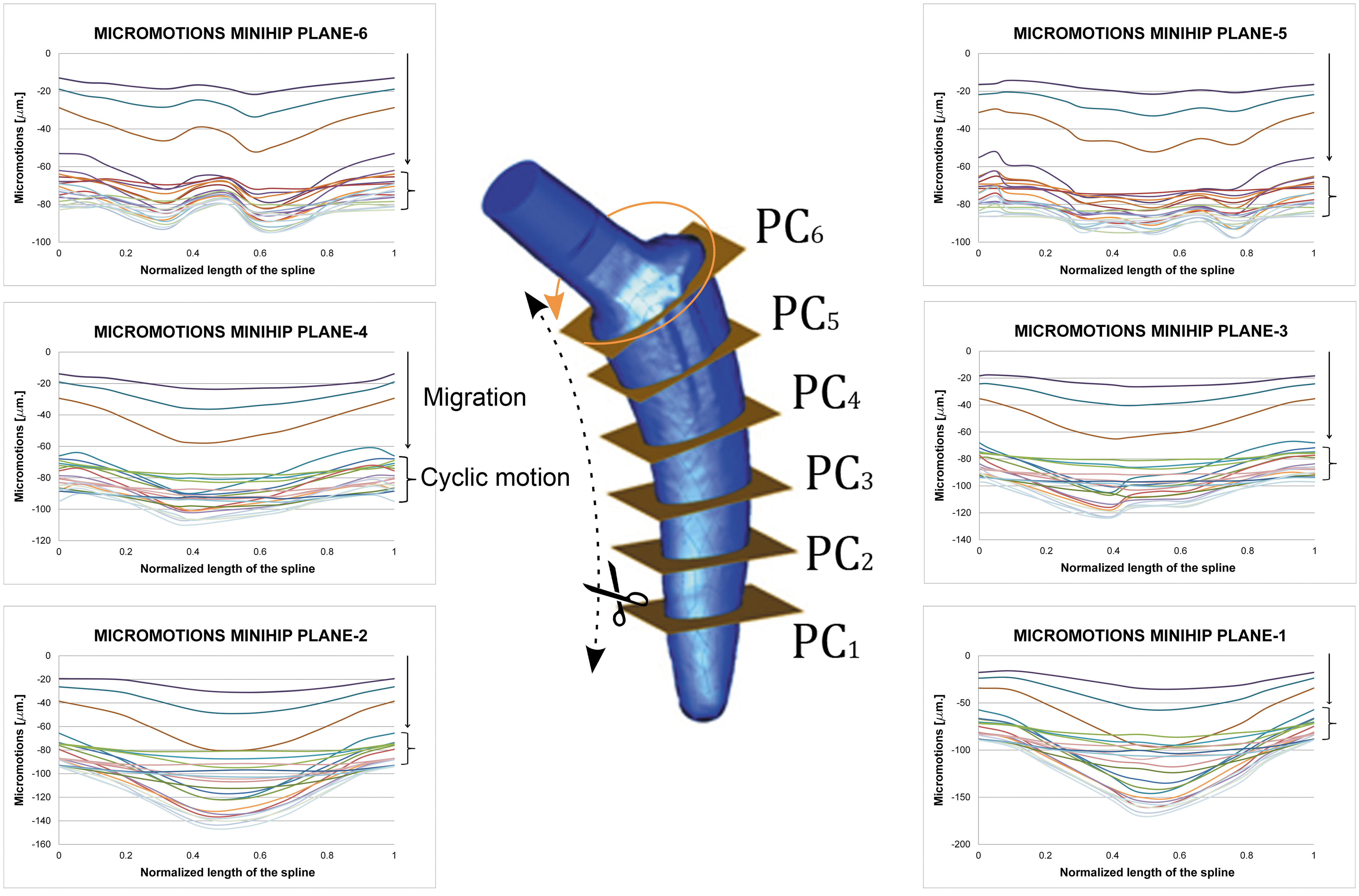

Supplement: S6 Fig — Normalized length in charts corresponds to the developed length for each plane cut starting at medial point in counter clock wise sense. Curves correspond to the different instants in gait cycle. (TIF) [file pone.0158411.s006.tif]

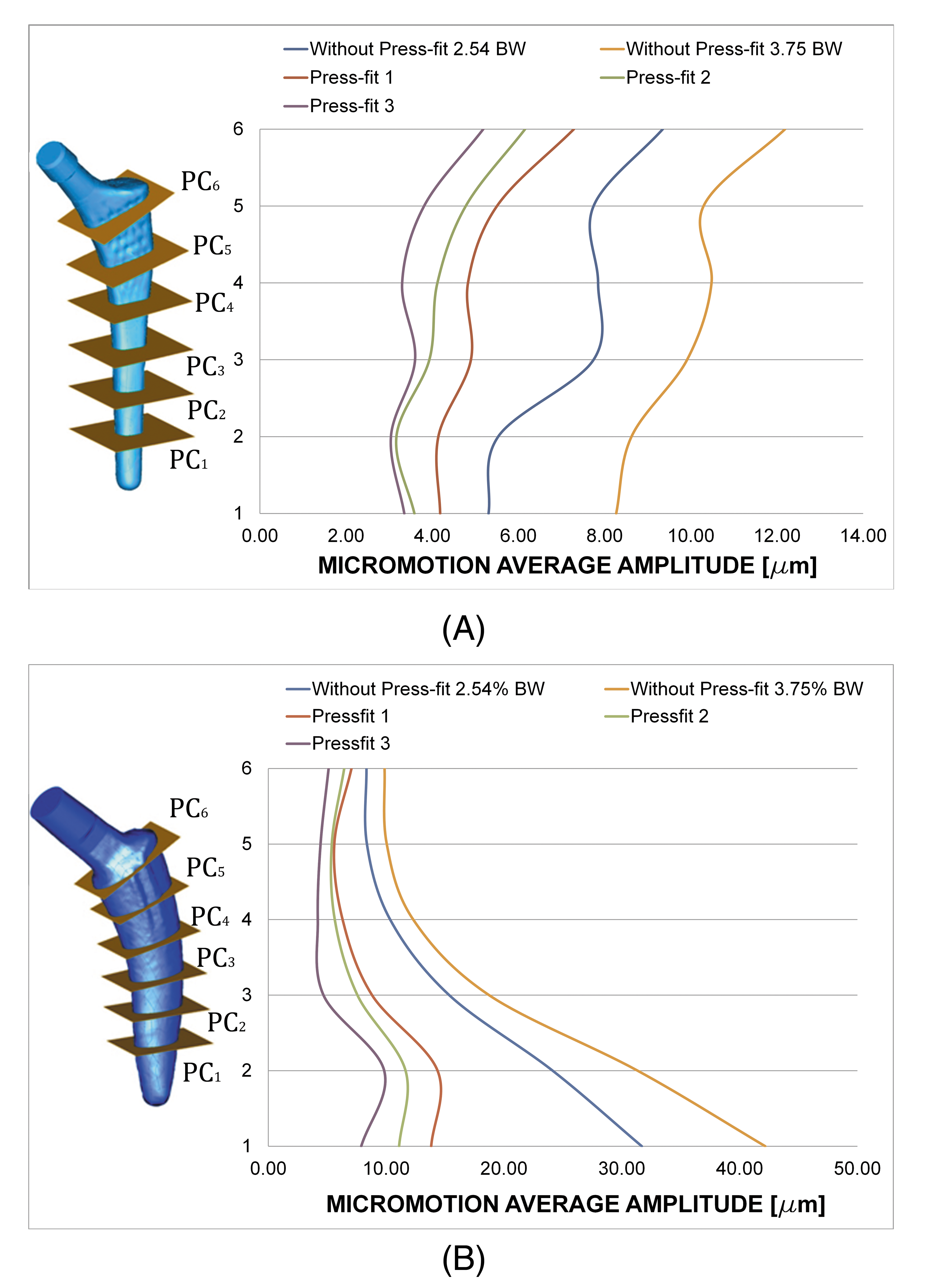

Supplement: S7 Fig — A and B. Average amplitude of micromotions for different press-fit levels and gait load cycle (friction coefficient μ = 0.5, Titanium alloy). (A) Linea stem. (B) Minihip stem. (TIF) [file pone.0158411.s007.tif]

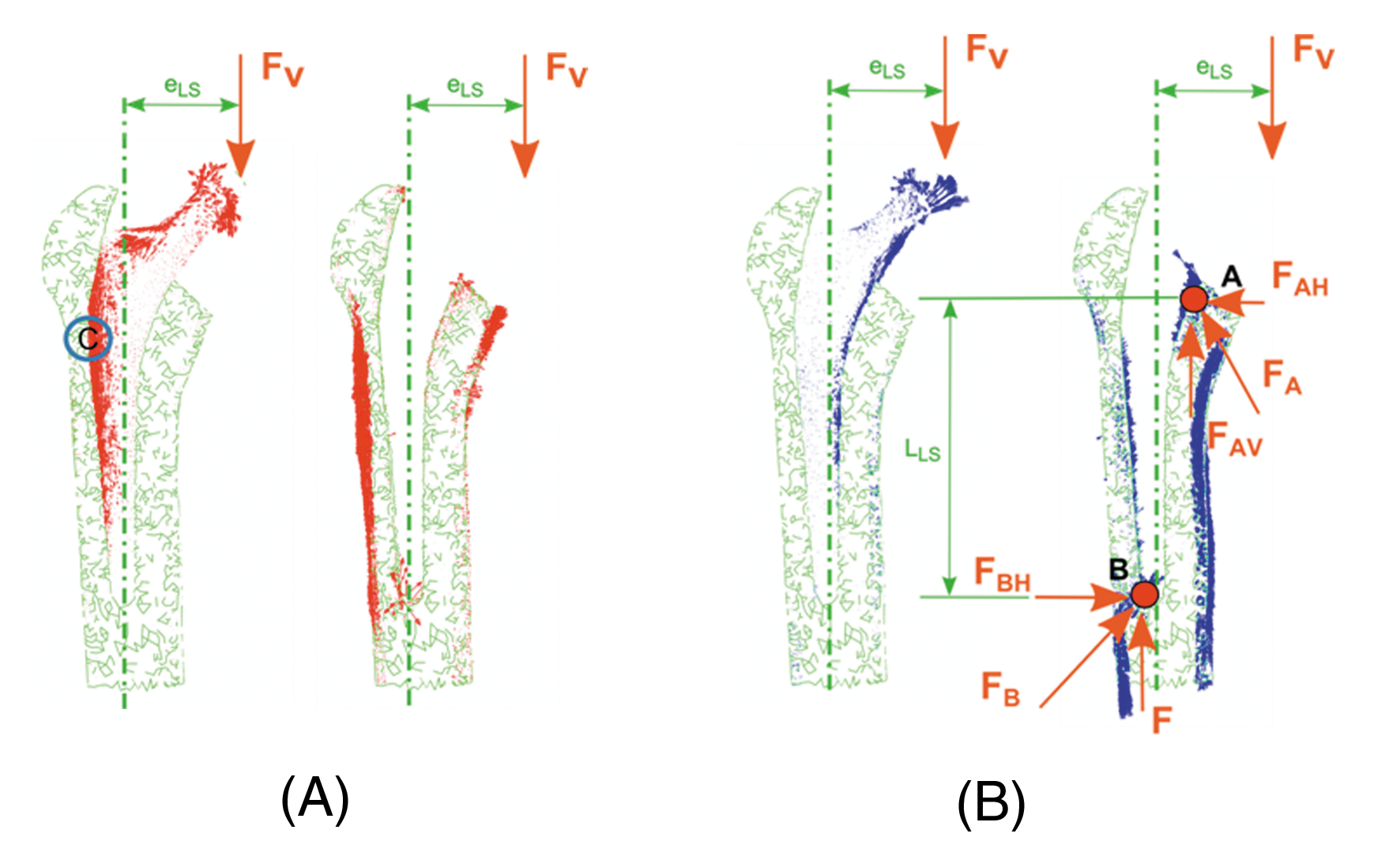

Supplement: S8 Fig — A and B. Coronal plane. View of principal flux stresses. Proximal femur with/without Linea stem. (A) Maximum principal stress. (B) Minimum principal stress. (TIF) [file pone.0158411.s008.tif]

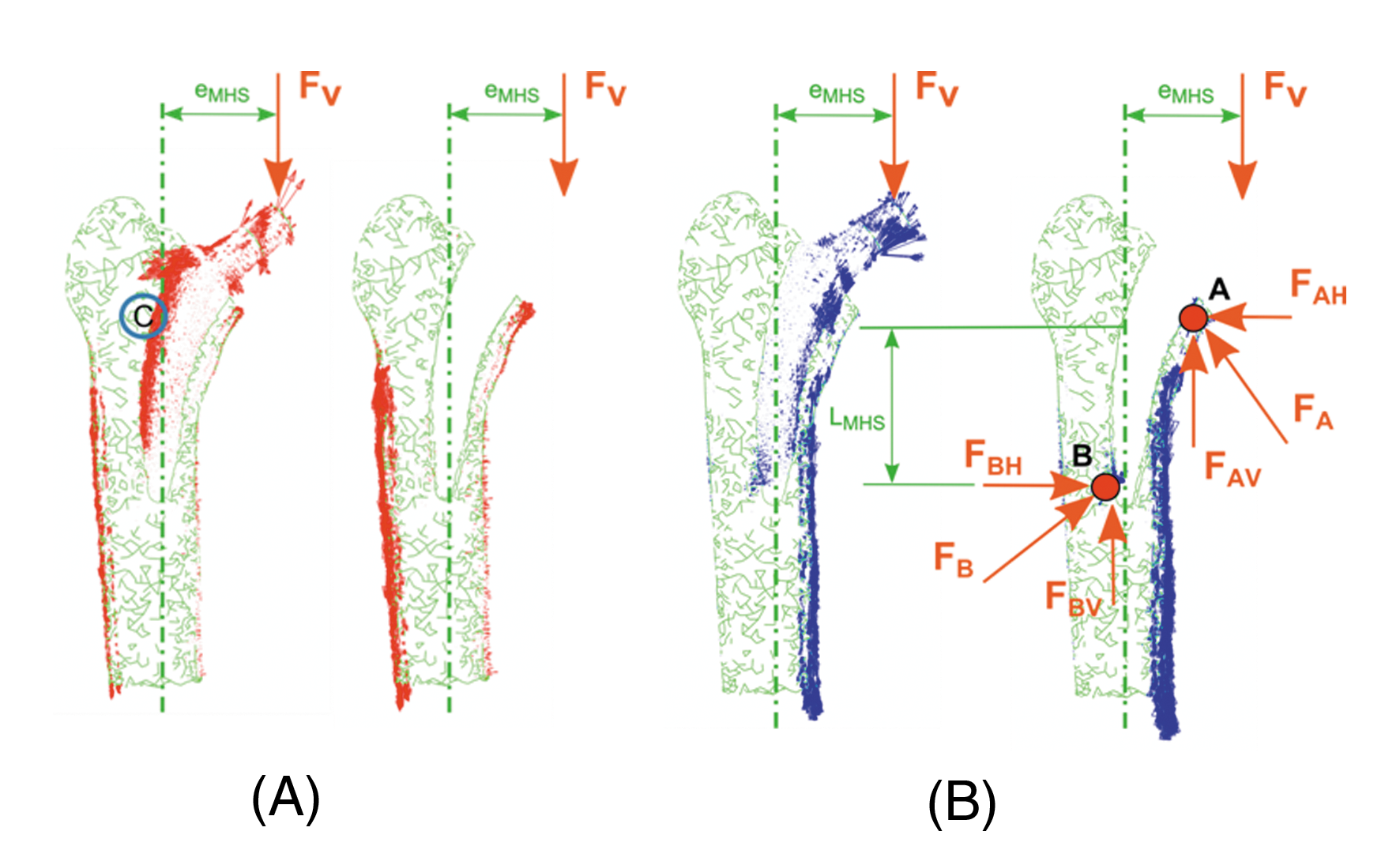

Supplement: S9 Fig — A and B. Coronal plane. View of principal flux stresses. Proximal femur with/without Minihip stem. (A) Maximum principal stress. (B) Minimum principal stress. (TIF) [file pone.0158411.s009.tif]

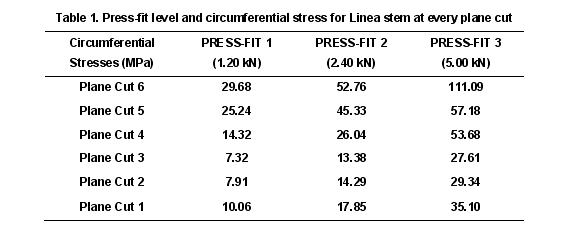

Supplement: S1 Table — (TIF) [file pone.0158411.s010.tif]

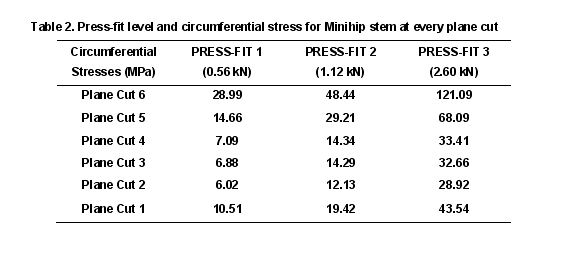

Supplement: S2 Table — (TIF) [file pone.0158411.s011.tif]
